# Unraveling the Binding Mode of Cyclic Adenosine–Inosine Monophosphate (cAIMP) to STING through Molecular Dynamics Simulations

**DOI:** 10.3390/molecules29112650

**Published:** 2024-06-04

**Authors:** Meiting Wang, Baoyi Fan, Wenfeng Lu, Ulf Ryde, Yuxiao Chang, Di Han, Jiarui Lu, Taigang Liu, Qinghe Gao, Changpo Chen, Yongtao Xu

**Affiliations:** 1 School of Medical Engineering & Henan International Joint Laboratory of Neural Information Analysis and Drug Intelligent Design, Xinxiang Medical University, Xinxiang 453003, China; wangmt@xxmu.edu.cn (M.W.); hd@xxmu.edu.cn (D.H.); lujiarui@xxmu.edu.cn (J.L.); liuttgg@163.com (T.L.); 2 Department of Computational Chemistry, Chemical Centre, Lund University, SE-221 00 Lund, Sweden; ulf.ryde@compchem.lu.se; 3 School of Pharmacy, Xinxiang Medical University, Xinxiang 453003, China; gao_qinghe@xxmu.edu.cn; 4 Henan Key Laboratory of Organic Functional Molecule and Drug Innovation, Key Laboratory of Green Chemical Media and Reactions of Ministry of Education, Collaborative Innovation Center of Henan Province for Green Manufacturing of Fine Chemicals, School of Chemistry and Chemical Engineering, Henan Normal University, Xinxiang 453007, China

**Keywords:** cAIMP, STING, binding mode, conformational change, molecular dynamics, free energy calculation

## Abstract

The stimulator of interferon genes (STING) plays a significant role in immune defense and protection against tumor proliferation. Many cyclic dinucleotide (CDN) analogues have been reported to regulate its activity, but the dynamic process involved when the ligands activate STING remains unclear. In this work, all-atom molecular dynamics simulations were performed to explore the binding mode between human STING (hSTING) and four cyclic adenosine–inosine monophosphate analogs (cAIMPs), as well as 2′,3′-cGMP-AMP (2′,3′-cGAMP). The results indicate that these cAIMPs adopt a U-shaped configuration within the binding pocket, forming extensive non-covalent interaction networks with hSTING. These interactions play a significant role in augmenting the binding, particularly in interactions with Tyr167, Arg238, Thr263, and Thr267. Additionally, the presence of hydrophobic interactions between the ligand and the receptor further contributes to the overall stability of the binding. In this work, the conformational changes in hSTING upon binding these cAIMPs were also studied and a significant tendency for hSTING to shift from open to closed state was observed after binding some of the cAIMP ligands.

## 1. Introduction

**Figure 1:**
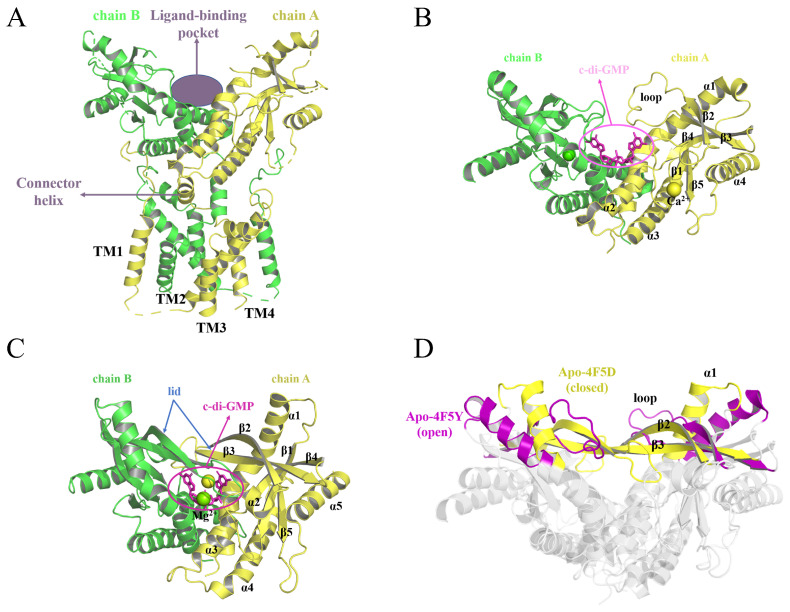
The X-ray crystal structures of hSTING. The A and B chains of hSTING are shown in yellow and green, respectively. (**A**) Structure of full-length hSTING (PDB code: 6NT5), (**B**) structure of hSTING in the open state (PDB code: 4F5Y), (**C**) structure of hSTING in the closed state (PDB code: 4F5D), and (**D**) superposition of hSTING structures in the open state (magenta) and closed state (yellow).

The stimulator of interferon genes (STING), also known as TMEM173, MPYS, EKIS, or MITA, is a transmembrane protein situated on the endoplasmic reticulum [1], which plays an important role in immune defense and protection against tumor proliferation by inducing the secretion of type I interferons (IFNs) and pro-inflammatory cytokines [2,3]. Crystallographic studies have shown that it is a dimer, composed of two monomers aligned in a V-shaped structure, consisting of 379 amino acid residues that form a transmembrane N-terminal structural domain and a globular C-terminal structural domain (CTD; cf. Figure 1A) [4,5,6,7]. The N-terminal domain is composed of four transmembrane helices, TM1-4, and a connector helix, while the C-terminal domain includes the ligand binding domain (LBD) and a C-terminal tail (CTT). Various investigations have revealed that STING activation or dimerization can be facilitated by the binding of cyclic dinucleotides [4,5,8,9]. The deep cleft that forms between the two monomers constitutes the ligand-binding site, which is largely hydrophobic [10]. The STING dimer exists primarily in two states, called “open” and “closed”. As is illustrated in Figure 1B–D, the main difference between these two states is that the closed state features a “lid” region formed by the β2β3 sheets, while the open state does not [11].

**Figure 2:**
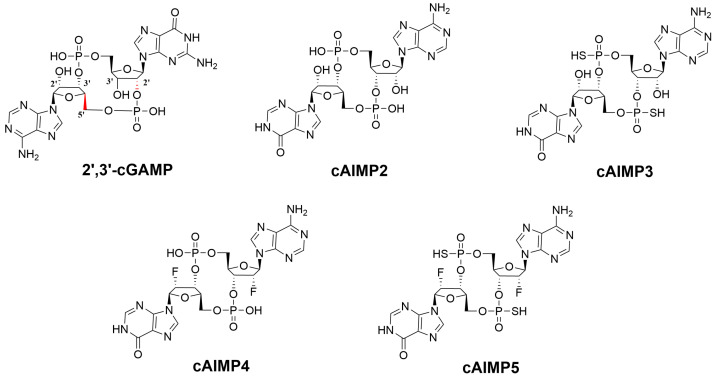
Structures of five STING agonists.

As a promising drug target, agonists targeting STING have been extensively investigated and some have entered various stages of clinical trials [12,13,14,15]. These agonists can be classified into two groups based on their molecular structure: cyclic dinucleotide agonists (CDNs) including c-di-AMP, c-di-GMP, 2′,3′-cGAMP, and 3′,3′-cGAMP, and non-cyclic dinucleotide agonists such as DMXAA, dimeric amidobenzimidazole (diABZI), SR-717, and MSA-2, etc.. The majority of these natural CDNs agonists are produced by bacteria, while 2′,3′-cGAMP, which is shown in Figure 2 and subsequently abbreviated as cGAMP, is synthesized by cyclic GMP-AMP synthase in mammals. Notably, cGAMP contains a unique 2′–5′ bond (marked in red), which may account for its high affinity for STING, in comparison to other CDNs [11,16,17]. Specifically, CDNs were initially identified as STING agonists. Unfortunately, due to the poor membrane permeability and metabolic instability, the biological activity and medical applications of the CDNs were limited.

Efforts to improve CDNs’ effectiveness include modifying phosphodiester bonds, ribose, and introducing F atoms [18,19,20,21]. Notably, ADU-S100, developed by Aduro and Novartis, has exhibited enhanced activity in inducing type I IFN production compared to cGAMP [22]. Similarly, compounds like IACS-8803 and IACS-8779 developed by MD Anderson are also being investigated for enhanced cellular permeability and activity [23]. Merck’s group designed MK-1454, which features modifications to both phosphodiester bonds and ribose moieties, resulting in improved cellular permeability [24,25].

Lioux’s group has also synthesized a series of novel cyclic adenosine–inosine monophosphate analogs (cAIMPs) by replacing the bases with hypoxanthine, in addition to modifying the phosphodiester bond and the ribose [26]. Among these compounds, the cAIMP2–5 agonists (Figure 2) have shown better activity than cGAMP in vitro. However, the detailed binding mechanism has not yet been fully understood. Despite numerous studies [26,27,28,29,30] exploring this field, the details of how cAIMP2–5 agonists interact with STING and trigger its downstream signaling pathway remain mysterious.

Some experiments have reported that the STING dimer undergoes a conformational change upon binding to cGAMP and diABZI [9,11,16,31]. These structural alterations are believed to be crucial for STING activation. In 2019, Shang et al. and Ergun et al. elucidated this mechanism [4,9]: the binding of cGAMP induced a 180° rotation of LBD relative to the TM domain and broke the internal crossing, which resulted in the formation of STING tetramers and higher-order oligomers by side-by-side packing. Ergun et al. discovered that the binding of cGAMP induced the closure of the STING dimer and the release of CTT, leading to the formation of the disulfide-linked polymers through the residue C148. The formation of these polymers is essential for subsequent reactions, including the phosphorylation of interferon regulatory factor 3 (IRF3), the indirect activation of the nuclear factor κ-light-chain-enhancer of activated B cells (NF-κB), and the production of type I IFN. However, Ramanjulu and colleagues demonstrated that, unlike cGAMP and DMXAA (5,6-dimethylxanthenone-4-acetic acid, Vadimezan), amidobenzimidazole-derived agonists can efficiently activate hSTING without the necessity for complete lid domain closure. In this case, hSTING may remain in a partially open conformation [32]. However, the complete elucidation of STING’s conformational changes is impeded by technological and experimental limitations. Although the mechanism has been studied by many scientists [33], as Boura’s research indicates, the binding of STING with agonists is highly complex, further complicating theoretical simulations [34].

In the current work, we focus on the binding of cAIMPs to hSTING. Molecular docking and molecular dynamics simulation were performed to explore the binding, including the binding mode, as well as the identification of key amino acid residues involved. In this study, both the open-hSTING (PDB code: 4F5Y) and closed-hSTING (PDB code: 4F5D) structures were utilized for all-atom molecular dynamics simulations, supplemented by free energy calculations, to elucidate the binding affinities of cAIMPs to STING in different states. We also studied conformational changes of hSTING upon binding to these agonists. The results indicate that all ligands adopt a U-shaped conformation within the binding pocket of hSTING, and hydrogen bonding and hydrophobic interactions between the ligands and some key residues (such as Tyr167, Arg238, Thr263, and Thr267) facilitate the binding. Furthermore, our calculations indicate that hSTING undergoes transitions from an open to a closed state upon binding three of the cAIMPs. These findings may offer valuable insights for the development of novel and potent CDN STING agonists in the future.

## 2. Results and Discussion

### 2.1. Binding Mode

The structure of cAIMP2 is similar to the reference compound cGAMP, besides the size of the ring consisting of the two ribose and phosphate moieties (cAIMP2 contains a 12-membered ring and cGAMP a 13-membered ring). The distinguishing feature between the two ligands lies in the constitution of the nucleobases, as illustrated in Figure 2. Specifically, cGAMP involves guanine and adenine, whereas cAIMP2 involves hypoxanthine and adenine. Among the four cAIMPs, different substituents were introduced in the ribose and the phosphate group. The hydroxyl groups (–OH) on the phosphate groups of cAIMP2 were replaced by sulfhydryl groups (–SH) in cAIMP3, which imparts hydrolysis and phosphatase resistance to cAIMP3. By replacing the free hydroxyl groups (–OH) on ribose with F atoms, cAIMP4 has greater lipophilicity than cAIMP2. In the case of cAIMP5, both phosphorothioate and fluorine modifications were introduced, which gives cAIMP5 better hydrolysis resistance and cell permeability.

#### 2.1.1. Binding to the Open State

**Figure 3:**
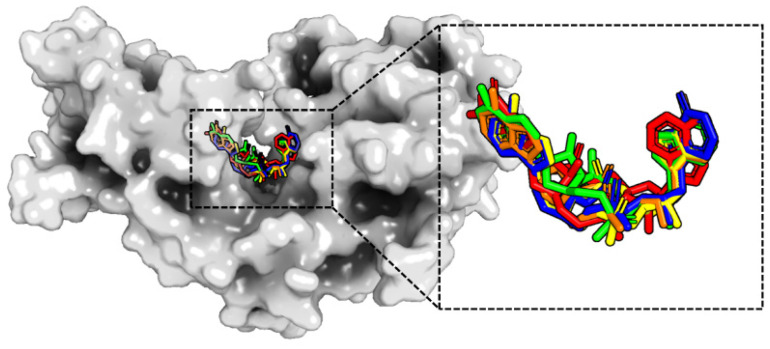
Surface map of hSTING in the open state and a structural superimposition of the five ligands after docking. The agonists are labeled with different colors: red for cGAMP; green for cAIMP2; blue for cAIMP3; yellow for cAIMP4; and orange for cAIMP5.

All five agonists were docked into the binding site of the hSTING dimer in the open state. As can be seen from Figure 3, all five molecules occupy the active pocket of hSTING with a well-defined U-shaped configuration. Upon structural superposition, the bases of the cAIMPs exhibited consistent alignment with a uniform orientation. However, the adenine of cGAMP was superimposed over the hypoxanthines of cAIMPs, while the guanine of cGAMP was observed to superimpose with the adenines of cAIMPs.

**Figure 4:**
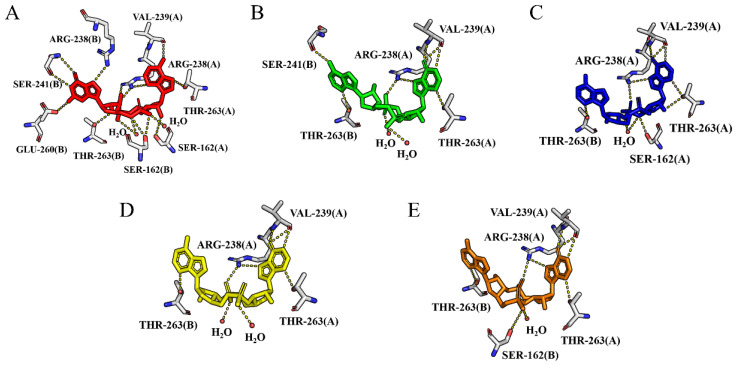
Hydrogen-bonding interaction networks of the five open systems after docking. The agonists are labeled with different colors: (**A**) red for cGAMP; (**B**) green for cAIMP2; (**C**) blue for cAIMP3; (**D**) yellow for cAIMP4; and (**E**) orange for cAIMP5.

The interactions between the ligands and hSTING are described in Figure S1 and Figure 4. Each of the basic groups of the agonists forms π–π stacking with one of the two copies of Tyr167 of the dimer. Meanwhile, the phosphate groups engage in interactions with residues Thr263, Arg238, Ser162, and Gly166. This is the main reason why the agonists maintain a U-shape in the pocket. Additionally, hydrophobic interactions between the agonists and residues Tyr163, Tyr167, Val239, Tyr240, Thr263, and Pro264 also enhance the binding stability, as shown in Figure S1.

**Figure 5:**
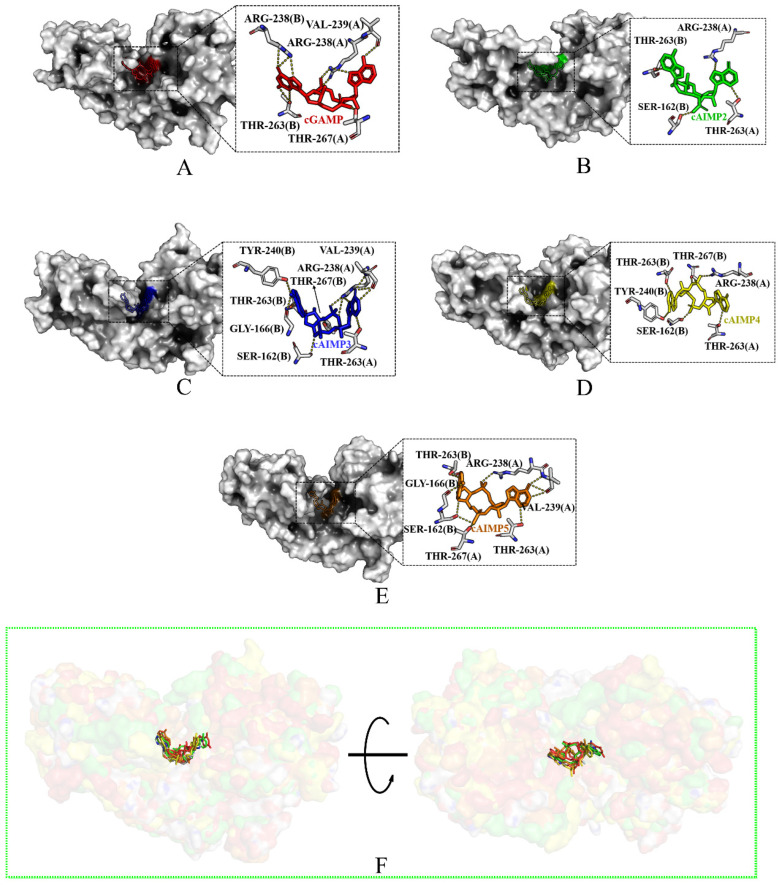
Surface maps of the five complexes systems in the open state (the protein residues are dimmed, and the ligands are represented by different colors) and hydrogen-bonding interaction networks for (**A**) cGAMP-4F5Y; (**B**) cAIMP2-4F5Y; (**C**) cAIMP3-4F5Y; (**D**) cAIMP4-4F5Y; (**E**) cAIMP5-4F5Y after 300 ns of MD simulations; and (**F**) superimposition of the five systems. Ligand colors are the same as in Figure 3.

The five complexes together with Apo-4F5Y (protein without any ligand) were subjected to 300 ns of MD simulations in triplicates. From the surface maps of the protein–ligand complexes (Figure 5A–E), it can be seen that all five ligands are wrapped by hSTING, thereby forming compact structures. A structural superposition (Figure 5F) shows that the cAIMPs penetrate the binding pocket deeper than cGAMP, indicating a potential for more stable binding interactions. This may contribute to the superior binding affinity of cAIMPs compared to cGAMP (which will be discussed below).

In order to investigate the stability of these systems, we measured the RMSD of these systems from the starting docked structure, and the results of the individual simulations are depicted in the Supporting Information Figures S2–S4 and in Table 1. Figures S2–S4 shows that the RMSD of all complexes is stable. Table 1 provides a summary of the mean and maximum RMSD values for all the complexes, as well as those of the ligands, over the final 20 ns of the simulations. The RMSD of cGAMP-4F5Y is 3.25 Å, whereas, it is 3.02 Å, 3.06 Å, 3.66 Å, and 3.27 Å for cAIMP2–5-4F5Y, respectively. All the data indicate that these five complexes and the Apo-4F5Y system are stable throughout the MD simulations.

Through the protein–ligand interactions (Figure 5A–E), a comprehensive understanding of the impact of various ligands on the stability of the hSTING structure and the binding mode between the protein and ligand can be obtained. Hydrogen bond occupancies were derived from the last 20 ns simulation, as is outlined in Table 2. As shown in Figure 5, the ligands were bound stably in the pocket and maintained the U-shaped conformation. Throughout the simulation, the majority of the hydrogen bonds persisted. For cGAMP-4F5Y, only Arg238A formed two strong hydrogen bonds with high occupancies, namely Arg238A-NH2···O11-cGAMP and Arg238A-NH1···O9-cGAMP, which indicate that Arg238 plays a critical role in the binding. On the other hand, the hydrogen bonds formed between cGAMP and Ser162, Ser241, as well as Glu260 disappeared during the simulation, which might weaken its binding to hSTING.

However, in the complexes of cAIMPs-4F5Y, five residues (Ser162, Arg238, Val239, Thr263, and Thr267) formed hydrogen bonds with relatively high occupancies. The importance of these residues is also reflected in the subsequent decomposition of binding free energy (Section 2.2). As delineated in Table 2 and Figure 4 and Figure 5, the hydrogen bonds between the four cAIMP molecules and Thr263 from both chains were consistently maintained throughout the simulation and exhibited varying degrees of reinforcement. This shows that Thr263 is crucial for sustaining the binding stability between the agonists and hSTING.

On the other hand, for the complexes of cAIMPs-4F5Y, various new hydrogen bonds were formed during the simulations. In particular, cAIMP2 and cAIMP4 formed robust hydrogen bonds with Ser162 (Ser162B-OG) in the complexes with 4F5Y, and the occupancies of these H-bonds are as high as 96% and 98%. In cAIMP5-4F5Y, two new hydrogen bonds were formed with Thr267A-OG1 and Arg238A-NH1, with occupancies of 30% and 25%, respectively. Based on the analysis of hydrogen bonds and their occupancies in each system, it seems likely that cAIMP3 and cAIMP5 exhibit a stronger interaction with hSTING in the open state compared to cGAMP. Meanwhile, the disappearance of hydrogen bonds between cAIMP2 and residues Val239 and Ser241 suggests a reduction in binding affinity.

#### 2.1.2. Binding to the Closed State

**Figure 6:**
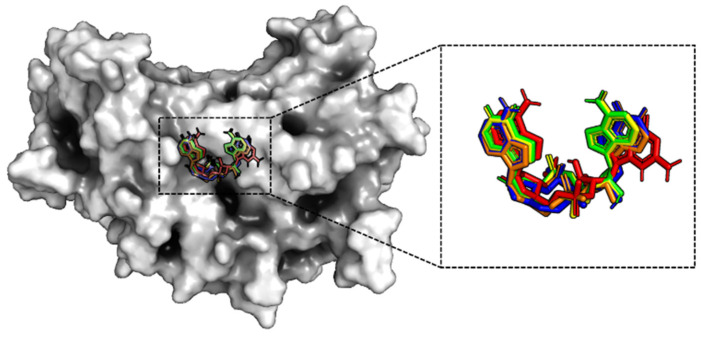
Surface map of hSTING in the closed state and a structural superposition of the five ligands after docking. (Red for cGAMP; green for cAIMP2; blue for cAIMP3; yellow for cAIMP4; and orange for cAIMP5.)

Molecular docking was applied to predict the structure of the complexes in the closed state, in the same way as that for the open state. A surface plot of protein with a structural superposition of the ligands is depicted in Figure 6. Clearly, the agonists are situated in the active pocket of hSTING in a U-shaped conformation. It is well-established that the structural pose of the ligand may differ based on the conformation of hSTING. Specifically, the U-shaped structure of the ligand bound in the closed state is more folded than that in the open state (compare Figure 3 and Figure 6). Additionally, the orientations of hypoxanthine and adenine in the four cAIMP ligands are not identical, in contrast to what was found for the open state. These conformations could be divided into two poses with opposite directions of bases. The bases of cAIMP2 superimpose well with those of cAIMP4, while the bases of cAIMP3 superimpose with those of cAIMP5. Notably, the adenine orientation of cGMAP is consistent with that in cAIMP3 and cAIMP5.

**Figure 7:**
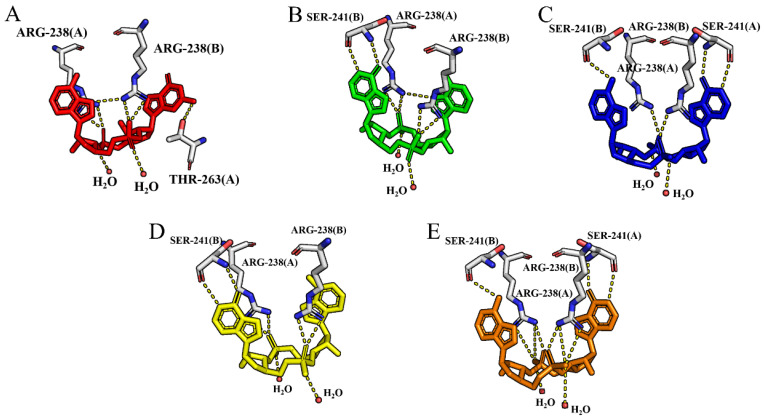
Hydrogen-bonding networks of the five closed systems after docking. The five agonists are labeled with different colors: (**A**) red for cGAMP; (**B**) green for cAIMP2; (**C**) blue for cAIMP3; (**D**) yellow for cAIMP4; and (**E**) orange for cAIMP5.

The 2D interactions between the agonists and hSTING are depicted in Figure S5, and the hydrogen-bonding interaction networks of these five complex systems are shown in Figure 7. From the docked structures, it is clear that hydrogen bonds between the ligand and Arg238 from both chains are present in all systems. Moreover, cAIMP3 and cAIMP5 formed hydrogen bonds with Ser241 from both chains, while cAIMP2 and cAIMP4 only formed hydrogen bonds with Ser241A. On the other hand, the hydrogen bond with Thr263 only existed in the cGAMP-4F5D complex. Similar to the open state, Tyr167 of the closed dimer also formed π–π stacking interactions with all ligands. There are also salt bridges between each ligand and Arg232A, as well as with two magnesium ions.

Next, 250 ns of MD simulations were carried out in triplicates for the five complexes constructed by molecular docking, while 100 ns of MD simulation was performed for Apo-4F5D as a reference. RMSD plots of these systems are depicted in Figures S6–S8, which show that all five complexes and Apo-4F5D are stable during the MD simulations with the RMSD mostly less than 3 Å for the complexes and less than 2 Å for the ligands. The mean and maximum RMSD of the last 20 ns of the simulations are summarized in Table S2. The RMSD of cGAMP-4F5D fluctuated around 2.26 Å, whereas the mean RMSD for cAIMP2–5-4F5D was 2.52, 2.24, 2.35, and 2.06 Å, respectively.

**Figure 8:**
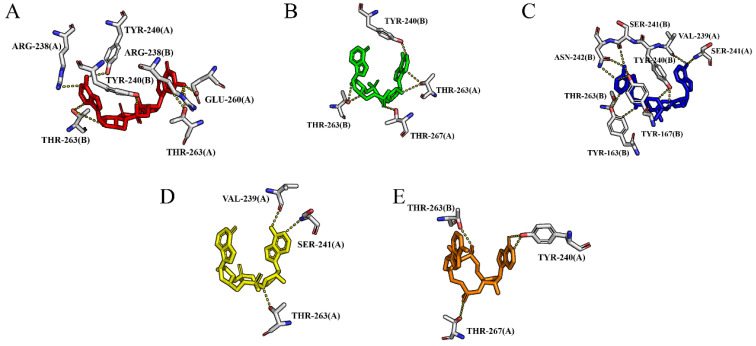
Hydrogen-bonding interactions for (**A**) cGAMP-4F5D; (**B**) cAIMP2-4F5D; (**C**) cAIMP3-4F5D; (**D**) cAIMP4-4F5D; and (**E**) cAIMP5-4F5D after 250 ns of MD simulations.

Likewise, the last frame was used to analyze the receptor–ligand interactions (Figure 8). The hydrogen bond occupancies summarized in Table 3 were obtained from the last 20 ns of the simulations. Compared to the open state, both cGAMP and cAIMP2 formed a more extensive network of hydrogen bonds with an increased number of residues, including Tyr240 and Thr263. Specifically, for cAIMP2-4F5D, three stable and highly occupied hydrogen bonds were identified: Thr267A-OG1···O7-cAIMP2 (92%), Thr263A-OG1^…^N3-cAIMP2 (82%), and Tyr240B-OH^…^N4-cAIMP2 (78%). In contrast, the number of stable hydrogen bonds between cAIMP3–5 and the closed hSTING decreased, especially for cAIMP5. Only one moderately occupied hydrogen bond, Thr267A-OG1···S1-cAIMP5 (45%), was observed, and its bond energy (−3.4 kcal/mol) was lower than that in the open system (Thr267A-OG1···S1-cAIMP5, −3.7 kcal/mol). More detailed information can be found in Table 3. In particular, for the cAIMP5-4F5D system, all hydrogen bonds formed with residues Ser241 and Arg238 during docking disappeared during the MD simulation, which might lead to a decrease in the binding ability.

### 2.2. Binding Free Energy

The results of molecular docking and experimental data [26,35] are summarized in Table 4. Clearly, the experimental data show that cAIMP2–5 (EC_50_: 6.4 μM, 10.6 μM, 0.7 μM, and 0.4 μM) are superior to cGAMP (19.6 μM) in the induction of type I IFN in human blood. Also, in the human immune cell line, cAIMP2–5 exhibits better activity than cGAMP in activating the pathways of IRF (EC_50_: 5.1 μM, 1.6 μM, 1.1 μM, and 0.3 μM versus 7.2 μM) and NF-κB (EC_50_: 15.9 μM, 7.8 μM, 15.4 μM, and 1.6 μM versus 39.1 μM). In the present work, the docking scores for cAIMPs also surpass that of cGAMP in both the open and closed state, which is consistent with the experimental results.

MM/GBSA were performed to estimate the binding affinities and the results are summarized in Table 4, Tables S3 and S4. Consistent with the experimental findings, the Δ*G*_bind_ of all the compounds cAIMP2–5 is significantly more negative than that of cGAMP for the open conformation. Particularly, the binding affinity of cAIMP3 (−48 kcal/mol) and cAIMP5 (−45 kcal/mol) is significantly higher than that of the reference compound (−34 kcal/mol). This underscores the superior binding capacities of cAIMP3 and cAIMP5 in the open state, with cAIMP3 emerging as the most promising candidate. As detailed in Section 2.1, the formation of numerous hydrogen bonds between cAIMP3 and cAIMP5 with hSTING plays a pivotal role in maintaining binding stability. Conversely, during the last 20 ns of simulation, cAIMP2 establishes fewer hydrogen bonds, resulting in a weaker binding affinity.

cGAMP exhibits a more negative binding affinity to hSTING in the closed state than in the open state, with a calculated Δ*G*_bind_ of −38 kcal/mol. It surpasses the binding affinities of cAIMP3 and cAIMP5, which are only −34 and −36 kcal/mol, respectively. Yet, it is less negative than that of cAIMP2 and cAIMP4, −49 and −40 kcal/mol. As elucidated in Section 2.1, cAIMP3 and cAIMP5 engage in few hydrogen bonding interactions with hSTING, and the occupancy levels are very low. It indicates that the interactions between cAIMP3 and cAIMP5 with hSTING are weak and unstable. The results suggest that cAIMP3 and cAIMP5 are more likely to bind to the open conformation of hSTING, while cGAMP, cGAMP2, and cAIMP4 are more inclined to associate with the closed conformation of hSTING.

**Figure 9:**
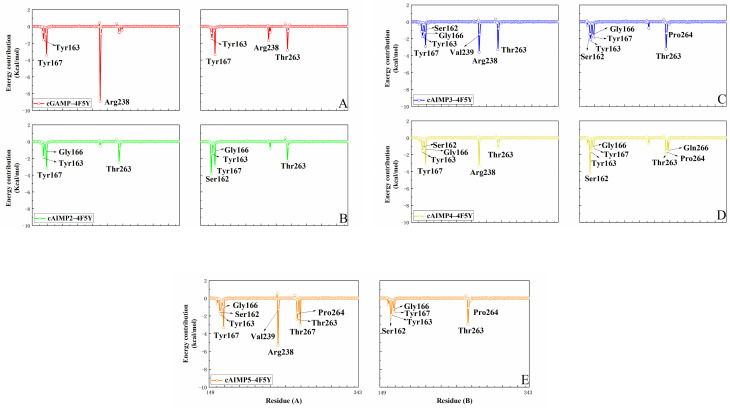
Per-residue decomposition of the MM/GBSA energies in the open state, (**A**) the cGAMP-4F5Y system, (**B**) the cAIMP2-4F5Y system, (**C**) the cAIMP3-4F5Y system, (**D**) the cAIMP4-4F5Y system, and (**E**) the cAIMP5-4F5Y system. Residues with energy contribution more negative than −1 kcal/mol are labelled. Figures on the left show residues from chain A, and those on the right from chain B.

**Figure 10:**
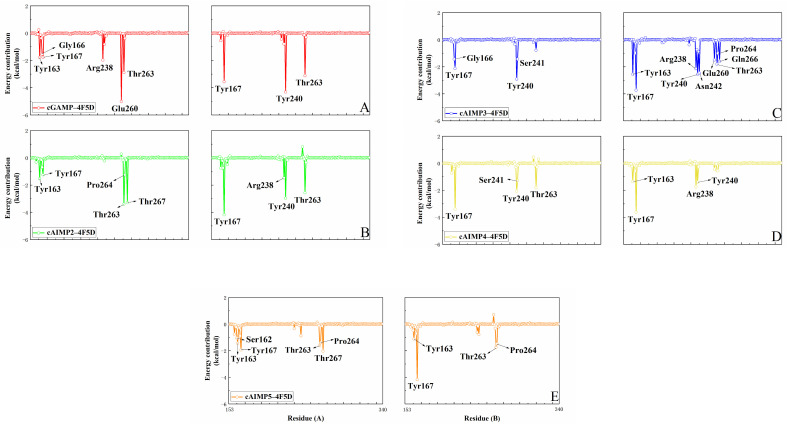
Per-residue decomposition of the MM/GBSA energies in the closed state, (**A**) the cGAMP-4F5D system, (**B**) the cAIMP2-4F5D system, (**C**) the cAIMP3-4F5D system, (**D**) the cAIMP4-4F5Dsystem, and (**E**) the cAIMP5-4F5D system. Residues with energy contribution more negative than −1 kcal/mol are labelled. Figures on the left show residues from chain A, and those on the right from chain B.

We also calculated free energy residue contributions by performing energy decomposition on the total binding free energy [36]. Figure 9 and Figure 10 show the results in both the open and closed states. Residues with energy contributions more negative than −1 kcal/mol have been highlighted. For the open systems (Figure 9), the key residues are primarily located at the bottom of the V-shaped structure of the hSTING protein (residues 162–167 and 263–267), as well as in a small portion of the loop region (238–239). Most of them form strong hydrogen bonds or hydrophobic interactions with the ligand (more details can be found in Section 2.1). In particular, both Tyr167A and Tyr167B exhibit moderate energy contributions ranging from −3.4 to −2.9 kcal/mol in all open complexes. This phenomenon can be attributed to the fact that Tyr167 forms π–π stacking interactions with all five ligands. In the case of cGAMP-4F5Y, Arg238A forms two highly robust hydrogen bonds, establishing it as the primary contributor, with a free energy contribution of −8.9 kcal/mol.

Compared to cGAMP, cAIMP2–5 binds more tightly to the bottom residues (including residues 162–167 and 263–267) of hSTING. Residues Ser162B, Thr263A, and Thr263B exhibit favorable energy contributions in the binding states (as detailed in Table S5). Multiple hydrogen bonds are formed between these residues and the ligands, with most of them displaying high hydrogen bond occupancies. Additionally, in cAIMP5-4F5Y, residues Arg238A and Thr267A also offer relatively high energy contributions of −5.3 kcal/mol and −3.0 kcal/mol, respectively. It can be seen from Figure 10 that these residues are mainly distributed in the bottom of the V-shaped structure of the hSTING protein (162–167 and 260–267), as well as a part of the β3 sheet (238–242) in the closed state. Similar to the results from the previous hydrogen bond analysis, residues forming stable hydrogen bonds with the ligands exhibit favorable energy contributions. Overall, Tyr163, Tyr167, Arg238, and Thr263 have prominent energy contributions to most of the systems in both the open and closed states.

### 2.3. Flexibility and Conformational Change

Many researchers [9,16,33,37,38,39] have reported that the STING dimer undergoes a conformational change upon the binding of effective ligands, and that the conformational change from the open to the closed state plays a crucial role in STING activation. In this work, the transition was also studied with MD simulations.

From the RSMD results listed in Table 1, it can be seen that the binding of cGAMP and cAIMPs increased the stability of hSTING, except for cAIMP4, which shows a slightly increased RMSD from 3.60 to 3.66 Å. Notably, the binding of cAIMP2 and cAIMP3 led to a decrease in RMSD by more than 0.5 Å.

**Figure 11:**
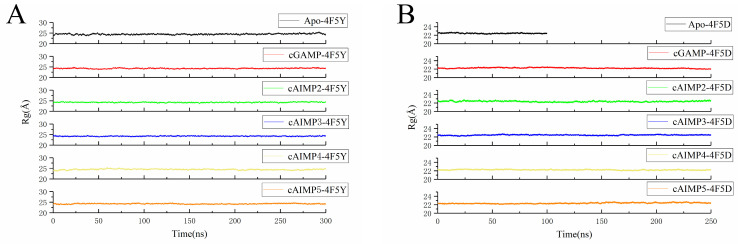
Radius of gyration of the open systems (**A**) and closed systems (**B**).

The structural compactness of the protein was examined by measuring the radius of gyration (Rg). The Rg results of the twelve systems are summarized in Figure 11 and Table 5. It can be observed that the average Rg values of all five complexes of the open hSTING are lower than that of Apo, and the Rg is lowest for cAIMP3 and cAIMP5. This indicates that during the simulation process, these two systems undergo more substantial conformational changes, making their structures more compact.

**Figure 12:**
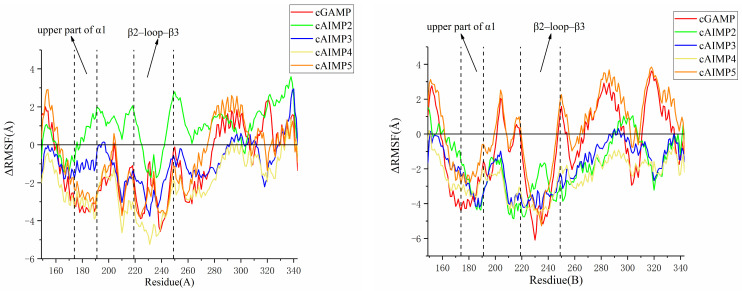
ΔRMSF between Apo hSTING and ligand-bound hSTING in the open state. The (**left figure**) shows residues from chain A, and the (**right figure**) residues from the chain B.

Moreover, the difference in RMSF (ΔRMSF) between Apo hSTING in the open state and ligand-bound hSTING was calculated to characterize the flexibility. The results are shown in Figure 12. It was found that the binding of almost all ligands reduced the flexibility of the upper part of the α1 helix (not cAIMP2) and β2-loop-β3 on the B chain. This suggests that these two regions have undergone significant conformational changes, showing a tendency towards the closed state.

**Figure 13:**
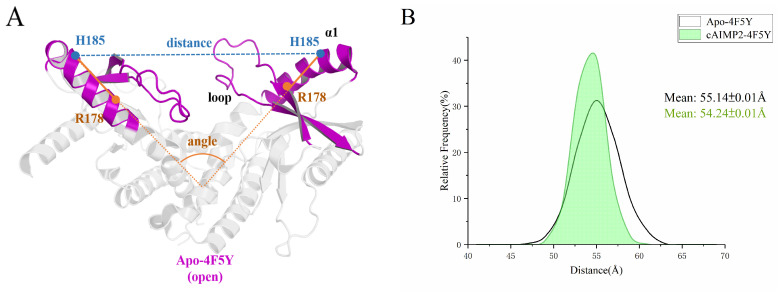

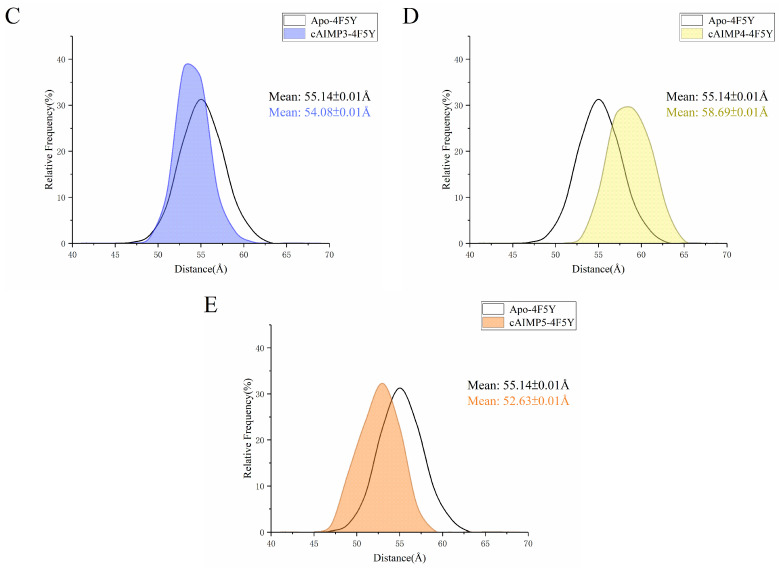
(**A**) Definition of the distance between His185 residues and the angle between the upper halves (178–185) of two α1 helices. (**B**–**E**) His185A–His185B distance distributions for all systems in the open state during the last 100 ns of MD simulations.

From the superposition of the open and closed states in Figure 1D, it can be seen that the differences are mainly in the β2-loop-β3 and the upper part of the α1 helix. Therefore, we also studied the conformational change of hSTING induced by binding to cAIMP2–5 by measuring the distance between the His185A and His185B residues in the α1 helices, as well as the angle between the upper halves of the two α1 helices (178–185; cf. Figure 13A) in the last 100 ns of MD simulation (more details can be found in Figures S9 and S10). Previous work [40] has shown that this distance is 32–38 Å in the closed state, whereas it is extended to 45–56 Å in the open state. In present work, the distances and angles of Apo-4F5Y were taken as the reference. Figure 13B–E presents the distance distributions of the systems in the open state during the last 100 ns of simulation. It is evident that the distance of the Apo-4F5Y fluctuates around 55.1 Å, which is greater than that of cAIMP2-4F5Y (54.2 Å), cAIMP3-4F5Y (54.1 Å), and cAIMP5-4F5Y (52.6 Å), but shorter than that of cAIMP4-4F5Y (58.7 Å). Compared to Apo-4F5Y, the distance distributions of cAIMP2-4F5Y, cAIMP3-4F5Y, and cAIMP5-4F5Y exhibit a significant leftward shift, especially for cAIMP5-4F5Y, showing a value of more than 2.5 Å.

**Figure 14:**
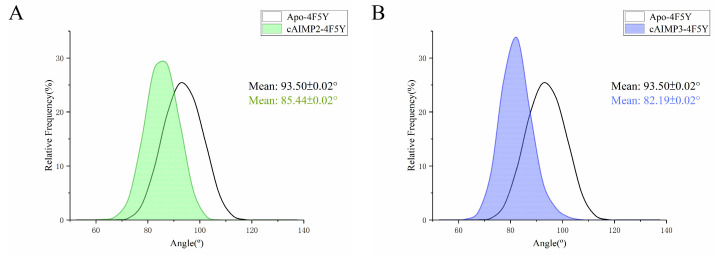

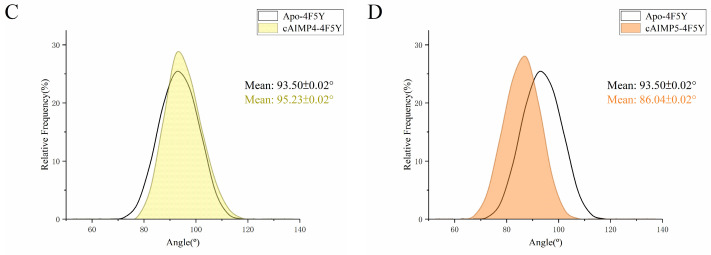
Angle distributions between the upper halves (residues 178–185) of two α1 helices for all systems in the open state during the last 100 ns of MD simulations. The reference Apo-4F5Y system is shown in white. (**A**) Green for the cAIMP2-4M5Y system; (**B**) Blue for the cAIMP3-4M5Y system; (**C**) Yellow for the cAIMP4-4M5Y system; (**D**) Orange for the cAIMP5-4M5Y system.

Moreover, the corresponding angle distributions are demonstrated in Figure 14A–D. Similar to the distance, compared to Apo-4F5Y (93.5°), the angles of cAIMP2-4F5Y (85.4°), cAIMP3-4F5Y (82.2°), and cAIMP5-4F5Y (86.0°) exhibit significant leftward shifts in all of the three systems by almost 10°. But the angle distributions of cAIMP4-4F5Y (95.2°) show a slight rightward trend. This result indicates that the binding of cAIMP2, cAIMP3, and cAIMP5 to hSTING can induce significant conformational changes of hSTING from the open state to closed state, although it did not reach a fully closed state within 300 ns simulations (Figure S11). However, for cAIMP4, such a transformation was not observed. On the contrary, binding cAIMP4 resulted in a slight increase in the openness of hSTING. Given the excellent activity of cAIMP4, it is possible that conformational transformation is not necessary for STING activation.

To visually elucidate the conformational changes induced by the binding of cAIMP2, cAIMP3, and cAIMP5, two representative conformations from the corresponding simulations were superposed, as illustrated in Figure S12. The discernible inward movement of the upper portion of the α1 helix and the loop region underscores the pronounced V-shaped configuration of the protein.

## 3. Theory and Methods

### 3.1. System Preparation and Simulation Details

The aim of the present study was to investigate the theoretical properties of hSTING agonists cAIMP2–5 from Lioux’s group. In addition, cGAMP was included as a reference. Two-dimensional structures of these five ligands were obtained from the PubChem database (https://pubchem.ncbi.nlm.nih.gov/, accessed on 1 May 2022). To explore the binding mode of these agonists to hSTING in different conformations, all the agonists were docked to two different hSTING structures: 4F5Y in the open state and 4F5D in the closed state [6,41]. Both of them were obtained from RCSB PDB (https://www.rcsb.org/, accessed on 22 April 2022). As a reference, both the open and closed conformations of hSTING without any ligand (called Apo) were also investigated. Consequently, a total of 12 systems were explored, namely Apo-4F5Y, cGAMP-4F5Y, cAIMP2-4F5Y, cAIMP3-4F5Y, cAIMP4-4F5Y, and cAIMP5-4F5Y for the open state, and Apo-4F5D, cGAMP-4F5D, cAIMP2-4F5D, cAIMP3-4F5D, cAIMP4-4F5D, and cAIMP5-4F5D for the closed state.

#### 3.1.1. Molecular Docking

All ligands were docked to hSTING using Molecular Operating Environment (MOE2019) [42]. For each ligand, geometry optimization and a conformational search were conducted using the QuickPrep module. Meanwhile, the downloaded protein structures were imported into MOE for structural optimization, including hydrogenation, dehydration, protonation, and energy minimization. Since the protein structures contained co-crystallized ligands (c-di-GMP, as shown in Figure 1), the position of the ligand was used to define the binding site for the docking. For each ligand, the docking process involved two steps. In the first step, the ligands were placed in the binding sites and optimized with the triangle matcher method, and the top 100 conformations were screened according to the London dG scoring function. In the second step, the induced-fit docking method was employed to further refine the top ten ligands from the first step with the GBVI/WSA dG (Generalized-Born Volume Integral/Weighted Surface area dG) force-field-based scoring function. From this, the best conformation was selected.

#### 3.1.2. Molecular Dynamics Simulation

Molecular dynamics (MDs) simulations were performed for the twelve systems (Section 3.1.1) with the Assisted Model Building with Energy Refinement (AMBER20) package [43,44]. The ff14SB force field [45] was employed to describe the proteins and the general AMBER force field (GAFF, Gromos Atlas of Functional Force-fields) [46] was applied for all the ligands. All Arg, Lys, Asp, and Glu residues were assumed to be charged. The protonation of His residues was determined based on the hydrogen bond network and solvent accessibility around the residues of interest (with a pH = 7.4 to mimic the physiological conditions. cf. Table S1). Partial charges for the ligands were obtained with the Austin Model 1-Bond Charge Corrected (AM1-BCC) method using Antechamber. Each system was solvated in an octahedral box containing Transferable Intermolecular Potential 3 Points (TIP3P) water molecules, ensuring a minimum distance of 10 Å between the ligand and the box edge. In addition, the systems were neutralized with sodium ions [47]. Topology and coordinate files were generated with the tleap module.

Each pre-prepared system was subjected to energy minimization by employing 5000 steps of steepest descent and 5000 steps of conjugated gradient minimization, with 500 kcal mol^−1^ Å^−2^ restraints on all heavy protein atoms, which was followed by 10,000 minimization steps without any restraint. Subsequently, the system was gently heated from 0 to 300 K with the force constant of the harmonic restraints reduced to 25 kcal mol^−1^ Å^−2^, and then equilibrated for 2 ns under the NPT ensemble. For all systems in the open state, a production run of 300 ns was executed with a time step of 2 fs at a constant temperature (300 K) and pressure (1 atm). For the closed and Apo states, a 250 ns production run was conducted for all systems, except for Apo-4F5D, for which 100 ns simulation was sufficient to ensure stability. The SHAKE algorithm [48] was employed to constrain all chemical bonds involving hydrogen atoms. Long-range electrostatic interactions were computed with the particle mesh Ewald (PME) method [49] along with periodic boundary conditions, and the Lennard-Jones interactions were cut off at a distance of 10 Å. All MD simulations were performed in triplicates.

### 3.2. Methods

#### 3.2.1. Trajectory Analysis

The root mean square deviation (RMSD) from the starting docked structure was used to explore the stability of the systems. Additionally, the RMSD values of ligands and Cα atoms of the protein were also calculated. To evaluate the compactness of the protein, the radius of gyration (Rg) was calculated. We calculated both the mass-weighted radius of gyration for all non-hydrogen atoms and the maximum value.

Furthermore, the root mean square fluctuations (RMSFs) during the last 20 ns of the simulations were calculated to analyze the conformational changes of hSTING. The RMSF determines the movement of each atom relative to its average position, and thus offers insights into the flexibility of the protein. Moreover, the distance between the Cα atoms of the His185 residues in the two α1 helices in each monomer of the protein and the angle between the upper halves (178–185) of the two α1 helices were also measured during the entire simulation. Alterations in the distance and angle correspond to changes in the protein’s conformation. Rg, RMSD, and RMSF were calculated with the CPPTRAJ module [50] in AmberTools21. A hydrogen bond analysis was also performed to check the stability of the hydrogen bonds.

#### 3.2.2. Binding Free Energy Calculation

The most widely employed free energy prediction method in drug design, Molecular Mechanics combined with Generalized Born and Surface Area solvation (MM/GBSA) [51,52], is utilized to predict the binding affinity between proteins and ligands in this study. In total, 3000 conformations from the last 60 ns of each trajectory were saved for free energy calculations [53]. The MMPBSA.py program [54] in AmberTools21 was utilized to perform free energy calculation for each system. With MM/GBSA, the binding free energy was obtained according to the following formula:

(1) $$∆G_{\mathrm{bind}}={\;G}_{\mathrm{com}}-(G_{\mathrm{pro}}+G_{\mathrm{lig}})$$

where $∆G_{\mathrm{bind}}$ is the total binding free energy. For each conformation, the binding free energy was computed for each molecular species, i.e., the complex ($G_{\mathrm{com}}$), the protein ($G_{\mathrm{pro}}$), and the ligand ($G_{\mathrm{lig}}$). The latter two terms were obtained by omitting the ligand or the protein from the snapshots from the simulation of the complex.

## 4. Conclusions

In this study, we employed all-atom molecular dynamics simulations to investigate the binding mechanisms of four cAIMPs with hSTING. By investigating the binding modes between these agonists and hSTING, stable binding was observed in both the open and closed states, characterized by the formation of numerous hydrogen bonds and hydrophobic interactions involving critical residues such as Tyr167, Arg238, and Thr263. The results reveal distinct affinities of these agonists towards different hSTING conformations. Compared to cGAMP, all these four cAIMPs show stronger affinities for the open state. Moreover, an evaluation of the dynamic changes in hSTING by molecular dynamics simulations indicates that the binding of cAIMP2, cAIMP3, and cAIMP5 induces significant conformational shifts from the open state to the closed state. But for cAIMP4, such a tendency was not observed. As emphasized by Watanab and colleagues, the full-length protein is critical for the study of STING. In the future, simulating the full-length protein in the membrane should be considered. Additionally, considering the characteristics of these cAIMPs and other CNDs, our new work on designing novel STING agonists using a fragment-based approach will be forthcoming.

## Figures and Tables

**Table 1:** RMSD for the open systems during the last 20 ns of the MD simulations. Reported precision is the standard error over a total of 20,000 snapshots.

System	Complex RMSD (Å)		Ligand RMSD (Å)	
	Mean	Max	Mean	Max
Apo-4F5Y	3.60 ± 0.00	5.05	-	-
cGAMP-4F5Y	3.25 ± 0.00	3.89	1.70 ± 0.00	2.03
cAIMP2-4F5Y	3.02 ± 0.00	3.67	0.51 ± 0.00	1.22
cAIMP3-4F5Y	3.06 ± 0.00	3.63	0.86 ± 0.00	1.64
cAIMP4-4F5Y	3.66 ± 0.00	4.53	1.46 ± 0.00	2.27
cAIMP5-4F5Y	3.27 ± 0.00	3.61	1.73 ± 0.00	2.39

**Table 2:** Hydrogen bonds formed during docking and in the MD simulations for the open state.

System	H-Bond		Docking			MD			
	Acceptor	Donor	Length (Å)	Angle (°)	Energy (kcal/mol)	Length (Å)	Angle (°)	Energy (kcal/mol)	Hydrogen Bond Occupancy
cGAMP-4F5Y	Lig-O11	Arg238A-NH2	2.1	136.4	−6.7	1.9	166.5	−8.5	90%
	Lig-O9	Arg238A-NH1	-	-	-	1.9	165.6	−8.2	83%
cAIMP2-4F5Y	Lig-O4	Ser162B-OG	-	-	-	1.7	161.2	−2.9	96%
	Lig-N3	Thr263B-OG1	2.6	159.9	−1.7	2.1	147.4	−1.7	29%
cAIMP3-4F5Y	Lig-O11	Thr263B-OG1	-	-	-	1.8	172.6	−1.6	51%
	Lig-N8	Thr263A-OG1	2.2	160.1	−2.0	2.1	165.4	−2.3	47%
	Lig-O9	Val239A-N	2.4	144.4	−1.3	2.0	155.5	−3.8	34%
	Lig-N3	Thr263B-OG1	2.3	141.4	−1.2	2.5	149.5	−2.4	32%
cAIMP4-4F5Y	Lig-O4	Ser162B-OG	-	-	-	1.6	159.0	−3.1	98%
	Lig-N3	Thr263B-OG1	2.3	144.8	−1.4	1.8	162.7	−1.8	36%
cAIMP5-4F5Y	Lig-N3	Thr263B-OG1	2.5	150.7	−1.4	2.0	169.8	−1.8	80%
	Lig-S1	Ser162B-OG	3.7	127.6	−0.6	2.2	157.5	−3.7	41%
	Lig-N8	Thr263A-OG1	2.2	159.9	−2.1	2.3	148.0	−1.3	34%
	Lig-O9	Val239A-N	2.2	150.2	−2.6	1.9	161.0	−2.6	32%
	Lig-S1	Thr267A-OG1	-	-	-	2.0	168.4	−3.7	30%
	Lig-O5	Arg238A-NH1	-	-	-	1.9	138.4	−6.4	25%

**Table 3:** Hydrogen bonds formed during docking and in the MD simulations for the closed state.

System	H-Bond		Docking			MD			
	Acceptor	Donor	Length (Å)	Angle (°)	Energy (kcal/mol)	Length (Å)	Angle (°)	Energy (kcal/mol)	Hydrogen Bond Occupancy
cGAMP-4F5D	Lig-O9	Tyr240B-OH	-	-	-	1.7	161.5	−4.4	97%
	Lig-O8	Thr263B-OG1	-	-	-	1.8	156.7	−1.2	38%
	Lig-N6	Thr263B-OG1	-	-	-	1.8	170.0	−1.9	27%
cAIMP2-4F5D	Lig-O7	Thr267A-OG1	-	-	-	1.8	162.9	−3.2	92%
	Lig-N3	Thr263A-OG1	-	-	-	1.9	156.7	−1.1	82%
	Lig-N4	Tyr240B-OH	-	-	-	1.8	160.5	−2.5	78%
cAIMP3-4F5D	Lig-N3	Tyr163B-OH	-	-	-	2.4	160.5	−3.2	37%
	Lig-O9	Ser241A-N	2.1	148.1	−2.2	1.9	164.1	−5.9	35%
cAIMP4-4F5D	Lig-O2	Thr263A-OG1	-	-	-	2.0	175.7	−1.1	44%
cAIMP5-4F5D	Lig-S1	Thr267A-OG1	-	-	-	2.1	148.1	−3.4	45%

**Table 4:** Experimental data ^a^, docking scores, and the average binding free energies of the five agonists. The reported binding free energies are the mean value and standard error (SE) obtained from three independent simulations.

Compound	THP1-Dual		Blood	Docking Score (kcal/mol)		Binding Free Energy (kcal/mol)	
	IRF EC_50_ (μM)	NF-κB EC_50_ (μM)	Type I IFN EC_50_ (μM)	Open	Closed	Open	Closed
cGAMP	7.2 ± 3.2	39.1 ± 22.6	19.6 ± 6.7	−8.85	−17.7	−33.7 ± 0.2	−37.6 ± 1.9
cAIMP2	5.1 ± 1.4	15.9 ± 4.1	6.4 ± 2.3	−9.18	−19.6	−34.5 ± 1.5	−49.6 ± 2.0
cAIMP3	1.6 ± 1.3	7.8 ± 1.3	10.6 ± 3.9	−9.69	−21.0	−48.1 ± 1.8	−34.0 ± 0.2
cAIMP4	1.1 ± 0.4	15.4 ± 3.2	0.7 ± 0.1	−9.92	−19.6	−33.9 ± 0.2	−40.2 ± 2.4
cAIMP5	0.3 ± 0.2	1.6 ± 0.3	0.4 ± 0.1	−9.72	−21.3	−45.1 ± 0.8	−35.9 ± 2.2

^a^ Ref. [26].

**Table 5:** Rg for all systems during the last 20 ns of the MD simulations. The reported uncertainties are standard errors calculated over 20,000 frames.

System	Rg (Å)		System	Rg (Å)	
	Mean	Max		Mean	Max
Apo-4F5Y	24.81 ± 0.00	25.66	Apo-4F5D	22.42 ± 0.00	22.64
cGAMP-4F5Y	24.41 ± 0.00	24.92	cGAMP-4F5D	22.06 ± 0.00	22.27
cAIMP2-4F5Y	24.40 ± 0.00	24.97	cAIMP2-4F5D	22.50 ± 0.00	22.86
cAIMP3-4F5Y	24.29 ± 0.00	24.82	cAIMP3-4F5D	22.47 ± 0.00	22.70
cAIMP4-4F5Y	24.54 ± 0.00	25.10	cAIMP4-4F5D	22.16 ± 0.00	22.42
cAIMP5-4F5Y	24.24 ± 0.00	24.71	cAIMP5-4F5D	22.42 ± 0.00	22.64

## Data Availability

The data presented in this study are available in article and Supplementary Materials.

## Supplementary Materials

The following supporting information can be downloaded at: https://www.mdpi.com/article/10.3390/molecules29112650/s1. Table S1: The protonation of His and HIE indicates that the nitrogen atom at position ε was protonated. Table S2: RMSD for the closed systems during the last 20 ns. Table S3: Binding free energies for the three simulations (60 ns) for open systems. Table S4: Binding free energies for the three simulations (60 ns) for closed systems. Table S5: Energy contributions of Ser162B, Thr263A and Thr263B in the cAIMPs-4F5Y system. Figure S1: 2D interaction diagrams between the five ligands and hSTING in the open state. (A) The cGAMP-4F5Y system, (B) the cAIMP2-4F5Y system, (C) the cAIMP3-4F5Y system, (D) the cAIMP4-4F5Y system, (E) the cAIMP5-4F5Y system. Figure S2: RMSD of the six systems in the open state from the first set of simulations: (A) the complexes; (B) the ligands. Figure S3: RMSD of the six systems in the open state from the second set of simulations: (A) the complexes; (B) the ligands. Figure S4: RMSD of the six systems in the open state from the third set of simulations: (A) the complexes; (B) the ligands. Figure S5: 2D interaction diagrams between five ligands and hSTING in the closed state. (A) The cGAMP-4F5D system, (B) The cAIMP2-4F5D system, (C) The cAIMP3-4F5D system, (D) The cAIMP4-4F5D system, (E) The cAIMP5-4F5D system. Figure S6: RMSD of the six systems in the closed state from the first set of simulations: (A) the complexes; (B) the ligands. Figure S7: RMSD of the six systems in the closed state from the second set of simulations: (A) the complexes; (B) the ligands. Figure S8: RMSD of the six systems in the closed state from the third set of simulations: (A) the complexes; (B) the ligands. Figure S9: Distance plots between His185A and His185B for all systems in open state during the MD simulations. The red dashed line in the figure represents the average distance of the Apo-4F5Y system throughout the MD simulation period. Figure S10: Angle plots between the upper halves (178–185) of two α1 helices for all systems in open state during the MD simulations. The red dashed line in the figure represents the average angle of the Apo-4F5Y system throughout the MD simulation period. Figure S11: His185A–His185B distance distributions for all systems in closed state during the MD simulations. Figure S12: Open-to-closed conformational changes during the MD simulations of (A) cAIMP2-4F5Y (purple color: the structure extracted at 51 ns, yellow color: the structure extracted at 209 ns), (B) cAIMP3-4F5Y (purple color: the structure extracted at 84 ns, yellow color: the structure extracted at 212 ns), and (C) cAIMP5-4F5Y (purple color: the structure extracted at 33 ns, yellow color: the structure extracted at 187 ns).
