# Supplementary material for: Unraveling the Binding Mode of Cyclic Adenosine–Inosine Monophosphate (cAIMP) to STING through Molecular Dynamics Simulations
Source: Molecules. 2024 Jun 4;29(11):2650. doi: 10.3390/molecules29112650 (PMC11173896; doi:10.3390/molecules29112650)
Supplement: Supplementary file 1 [file molecules-29-02650-s001.zip › molecules-3014726-supplementary.pdf]

Supporting information for: Unraveling the Binding Model of Cyclic Adenosine–Inosine Monophosphate (cAIMP) to STING through Molecular Dynamics Simulations

Meiting Wang [1,2,†], Baoyi Fan [1,†], Wenfeng Lu [1], Yuxiao Chang [1], Di Han [1], Jiarui Lu [1], Taigang Liu [1], Qinghe Gao [3], Changpo Chen [4], Ulf Ryde[2,*] and Yongtao Xu [1,*]

1 School of Medical Engineering & Henan International Joint Laboratory of Neural Information Analysis and Drug Intelligent Design, Xinxiang Medical University, Xinxiang 453003, China

2 Department of Computational Chemistry, Chemical Centre, Lund University, SE-221 00 Lund, Sweden

3 School of Pharmacy, Xinxiang Medical University, Xinxiang 453003, China

4 Henan Key Laboratory of Organic Functional Molecule and Drug Innovation, Key Laboratory of Green Chemical Media and Reactions of Ministry of Education, Collaborative Innovation Center of Henan Province for Green Manufacturing of Fine Chemicals, School of Chemistry and Chemical Engineering, Henan Normal University, Xinxiang, Henan 453007, China

* Correspondence: ulf.ryde@compchem.lu.se & yxu@xxmu.edu.cn

† These authors contributed equally to this work.

Table S1. The protonation of His and HIE indicates that the nitrogen atom at position ε was protonated.

**4F5Y**	HIE: 157A, 185A, 332A, 157B, 185B, 332B
**4F5D**	HIE: 157A, 185A, 332A, 157B, 185B, 332B

Table S2. RMSD for the closed systems during the last 20 ns.

System	Complex RMSD (Å)		Ligand RMSD (Å)	
	**Mean**	**Max**	**Mean**	**Max**
**Apo-4F5D**	2.52±0.00	2.97	-	-
**cGAMP-4F5D**	2.26±0.00	2.66	0.64±0.00	0.94
**cAIMP2-4F5D**	2.52±0.00	2.86	0.61±0.00	1.32
**cAIMP3-4F5D**	2.24±0.00	2.62	0.75±0.00	1.17
**cAIMP4-4F5D**	2.35±0.00	2.74	0.81±0.00	1.37
**cAIMP5-4F5D**	2.06±0.00	2.41	1.29±0.00	1.95

Table S3. Binding free energies for the three simulations (60 ns) for open systems.

System	Binding free energy (kcal/mol)			
	**1st**	**2nd**	**3rd**	**Ave**

**cGAMP-4F5Y**	-33.50±0.15	-33.49±0.17	-34.05±0.12	-33.68±0.19
**cAIMP2-4F5Y**	-36.64±0.16	-31.48±0.10	-35.24±0.10	-34.45±1.54
**cAIMP3-4F5Y**	-44.89±0.17	-48.49±0.08	-50.94±0.09	-48.11±1.76
**cAIMP4-4F5Y**	-33.41±0.13	-34.08±0.10	-34.09±0.09	-33.86±0.23
**cAIMP5-4F5Y**	-46.61±0.14	-44.51±0.12	-44.25±0.12	-45.12±0.75

Table S4. Binding free energies for the three simulations (60 ns) for closed systems.

**System**	**Binding free energy (kcal/mol)**			
	**1st**	**2nd**	**3rd**	**Ave**
**cGAMP-4F5D**	-37.18±0.15	-41.11±0.14	-34.59±0.11	-37.63±1.90
**cAIMP2-4F5D**	-45.62±0.18	-51.63±0.11	-51.60±0.11	-49.62±2.00
**cAIMP3-4F5D**	-33.65±0.15	-34.39±0.10	-33.88±0.12	-33.97±0.22
**cAIMP4-4F5D**	-38.56±0.11	-44.97±0.07	-37.19±0.12	-40.24±2.40
**cAIMP5-4F5D**	-32.74±0.10	-40.12±0.08	-34.76±0.08	-35.87±2.20

Table S5. Energy contributions of Ser162B, Thr263A and Thr263B in the cAIMPs-4F5Y system.

**System**	**Energy contribution (kcal/mol)**		
	**Ser162B**	**Thr263A**	**Thr263B**
**cAIMP2-4F5Y**	-3.83	-2.50	-2.29
**cAIMP3-4F5Y**	-1.43	-3.29	-3.20
**cAIMP4-4F5Y**	-4.20	-1.20	-1.94
**cAIMP5-4F5Y**	-2.15	-2.43	-2.94

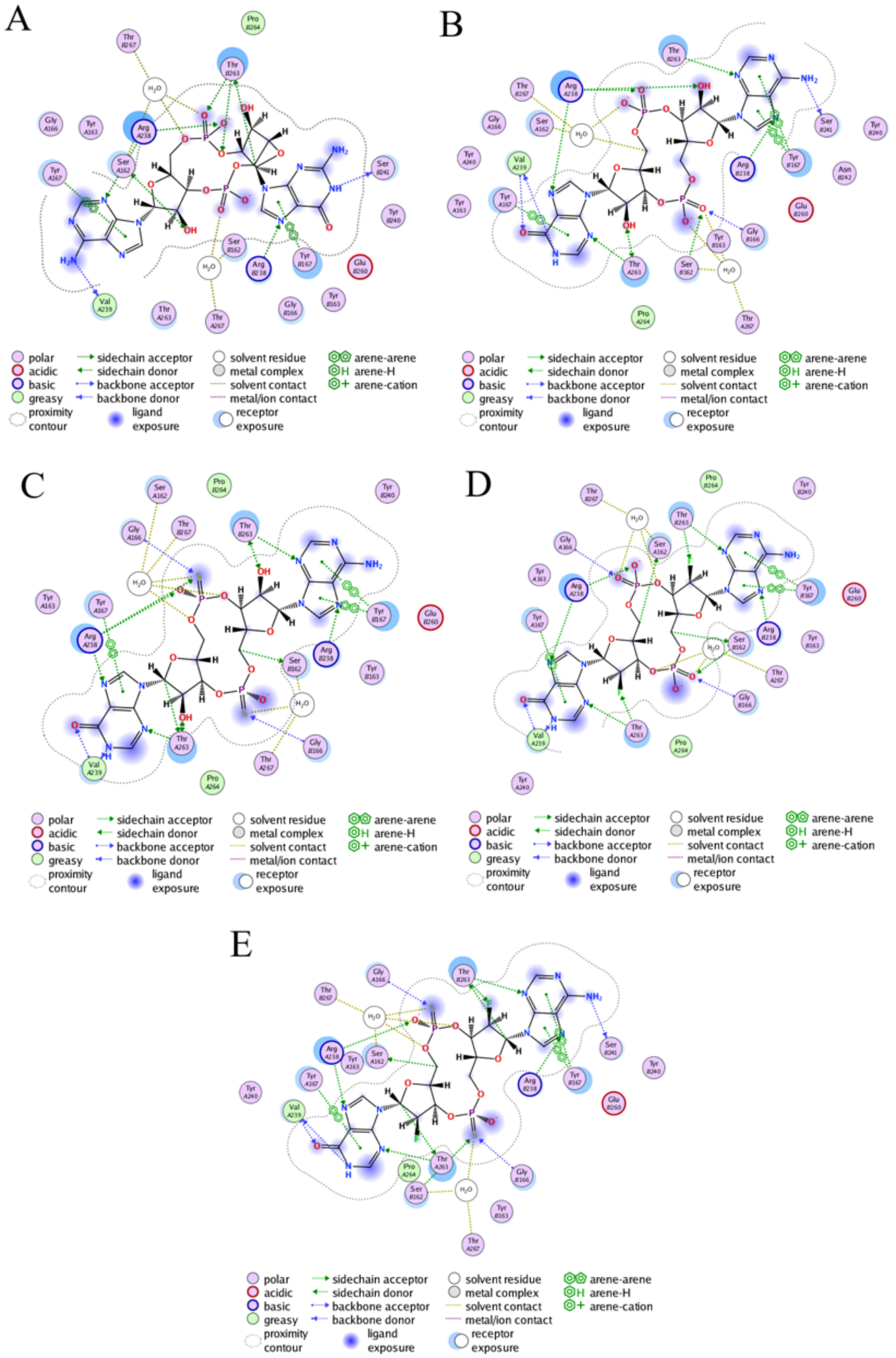

Figure S1. 2D interaction diagrams between the five ligands and hSTING in the open state. (A) The cGAMP-4F5Y system, (B) the cAIMP2-4F5Y system, (C) the cAIMP3-4F5Y system, (D) the cAIMP4-4F5Y system, (E) the cAIMP5-4F5Y system.

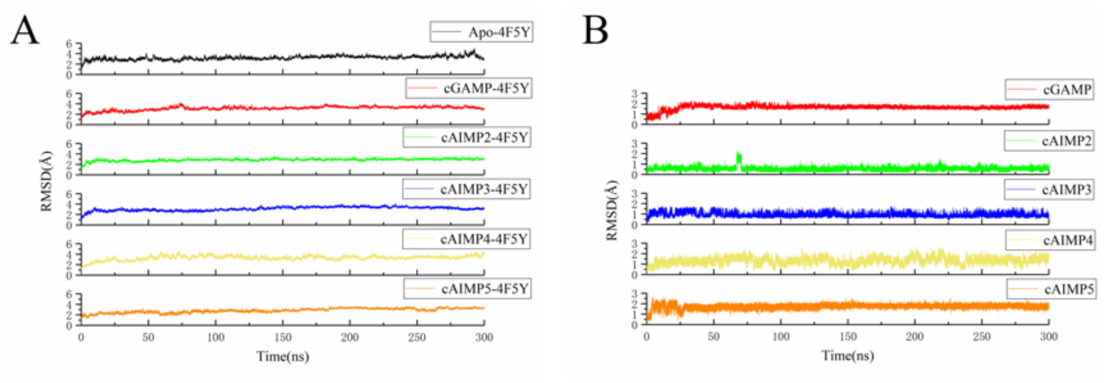

Figure S2. RMSD of the six systems in the open state from the first set of simulations: (A) the complexes; (B) the ligands.

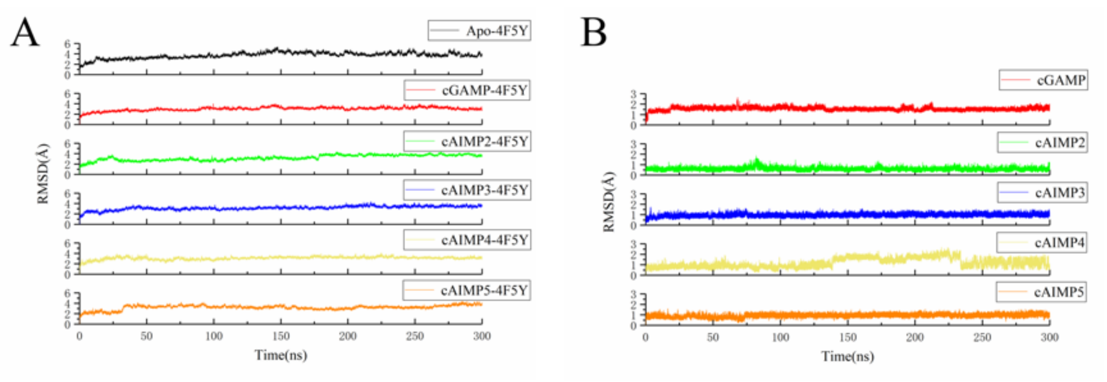

Figure S3. RMSD of the six systems in the open state from the second set of simulations: (A) the complexes; (B) the ligands.

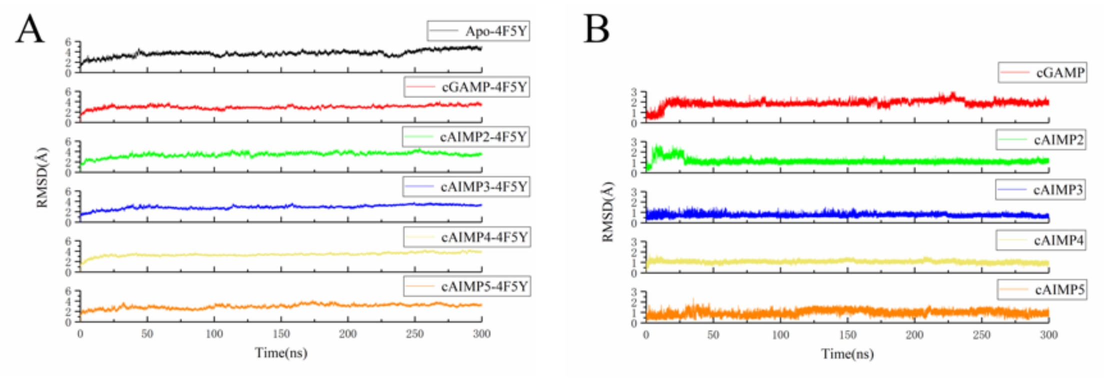

Figure S4. RMSD of the six systems in the open state from the third set of simulations: (A) the complexes; (B) the ligands.

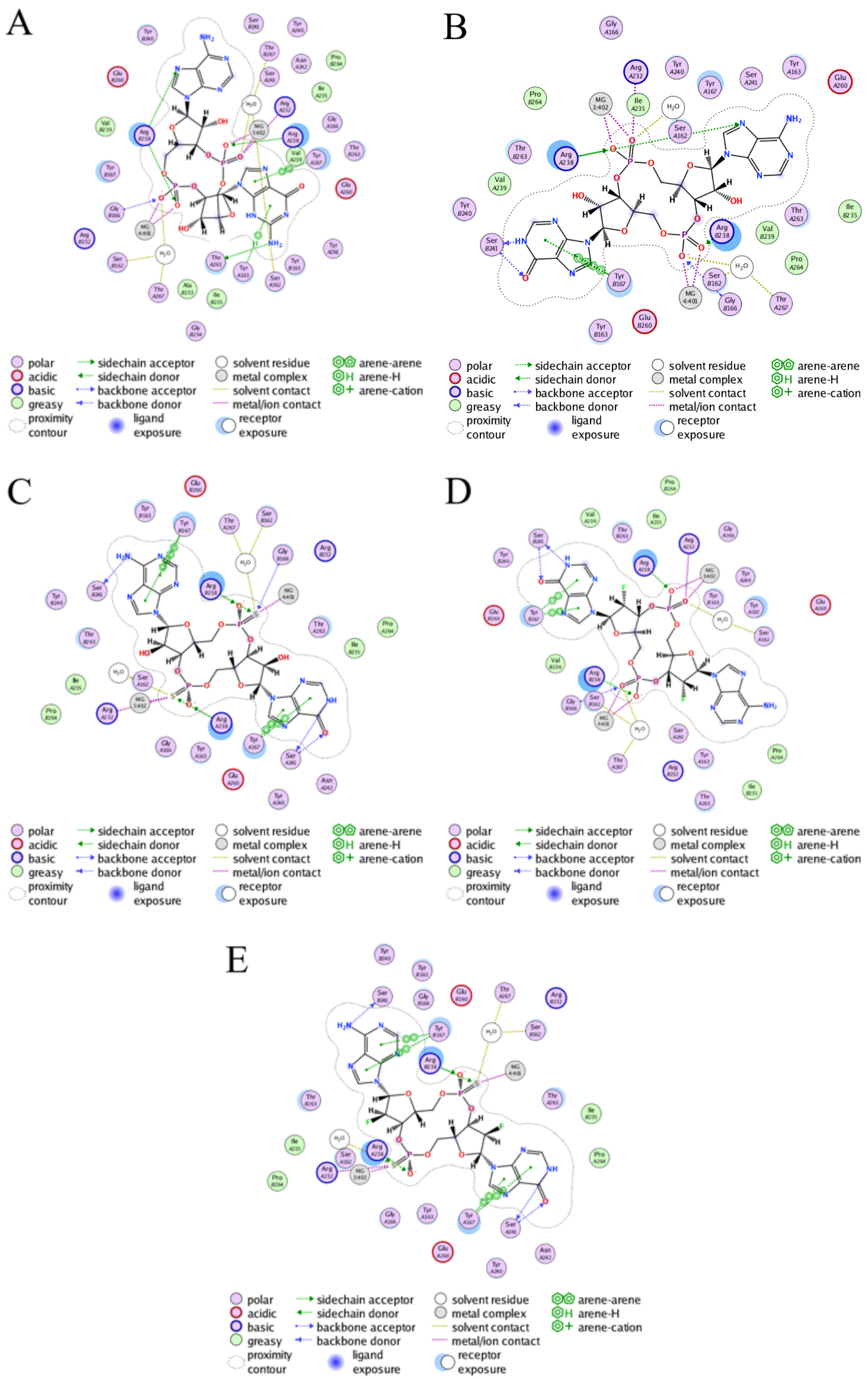

Figure S5. 2D interaction diagrams between five ligands and hSTING in the closed state. (A) The cGAMP-4F5D system, (B) The cAIMP2-4F5D system, (C) The cAIMP3-4F5D system, (D) The cAIMP4-4F5D system, (E) The cAIMP5-4F5D system.

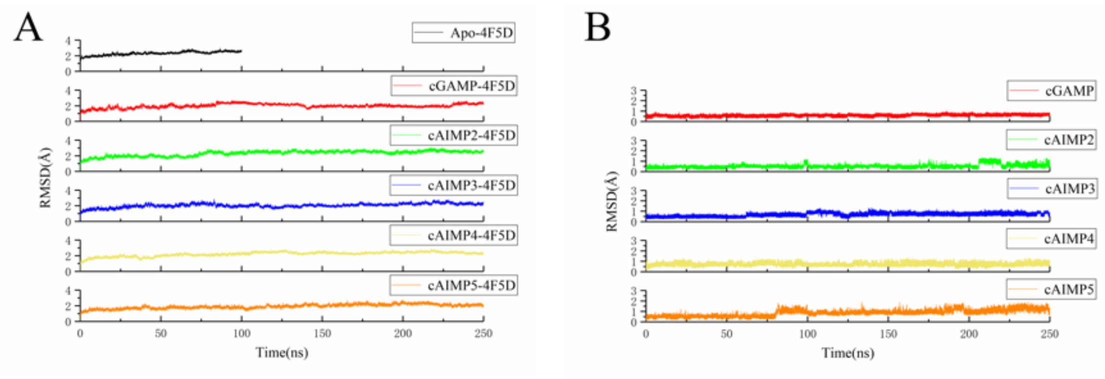

Figure S6. RMSD of the six systems in the closed state from the first set of simulations: (A) the complexes; (B) the ligands.

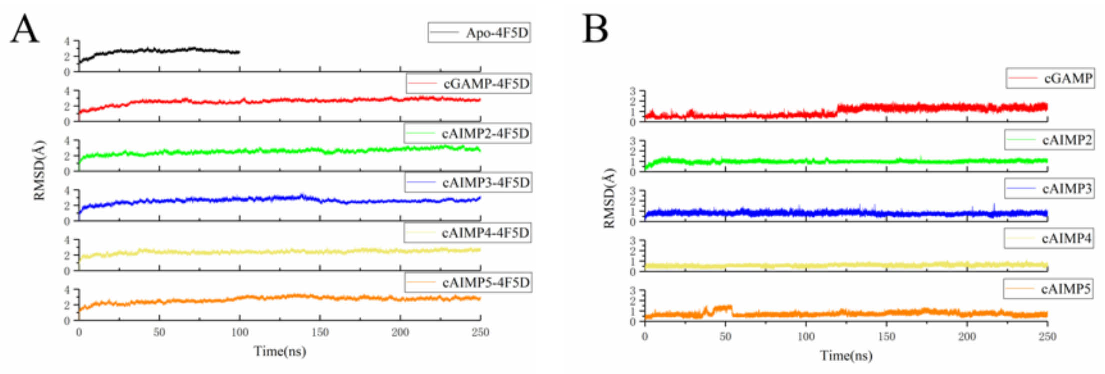

Figure S7. RMSD of the six systems in the closed state from the second set of simulations: (A) the complexes; (B) the ligands.

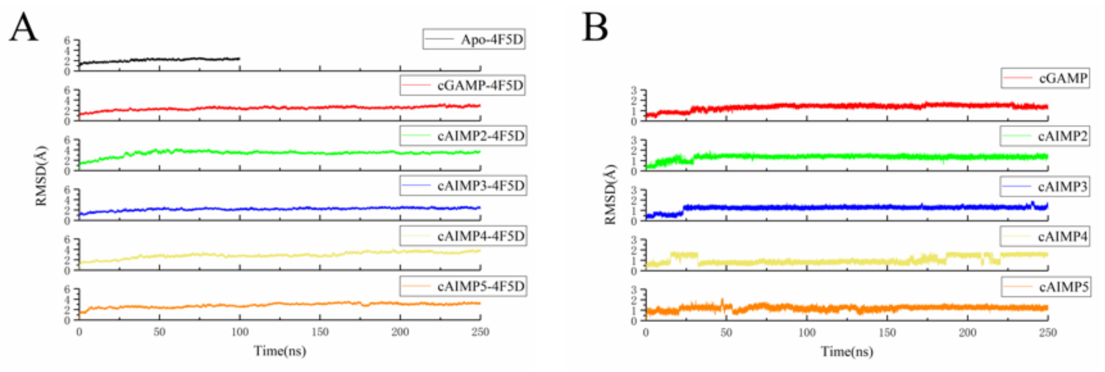

Figure S8. RMSD of the six systems in the closed state from the third set of simulations: (A) the complexes; (B) the ligands.

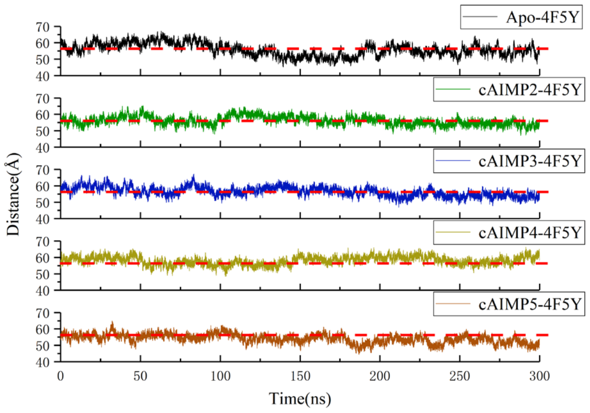

Figure S9. Distance plots between His185A and His185B for all systems in open state during the MD simulations. The red dashed line in the figure represents the average distance of the Apo-4F5Y system throughout the MD simulation period.

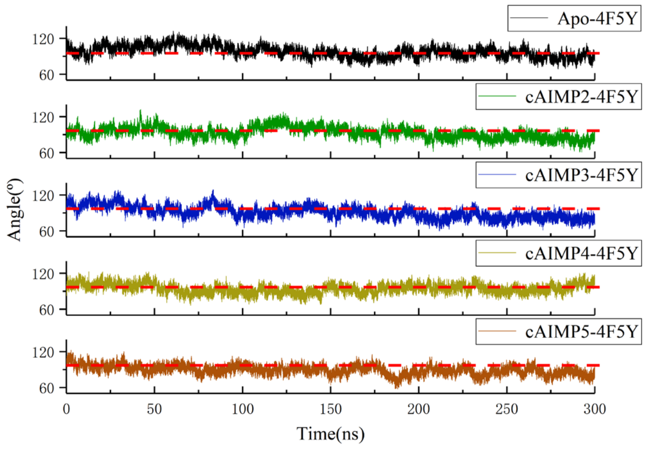

Figure S10. Angle plots between the upper halves (178–185) of two α1 helices for all systems in open state during the MD simulations. The red dashed line in the figure represents the average angle of the Apo-4F5Y system throughout the MD simulation period.

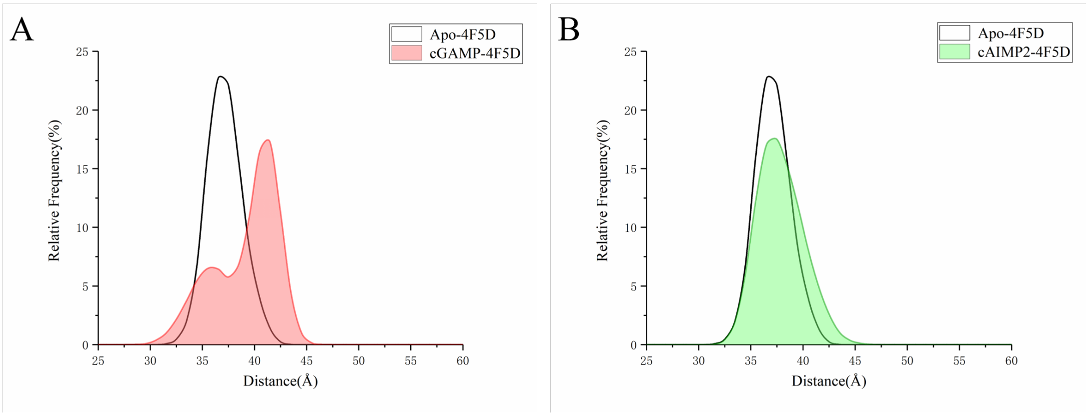

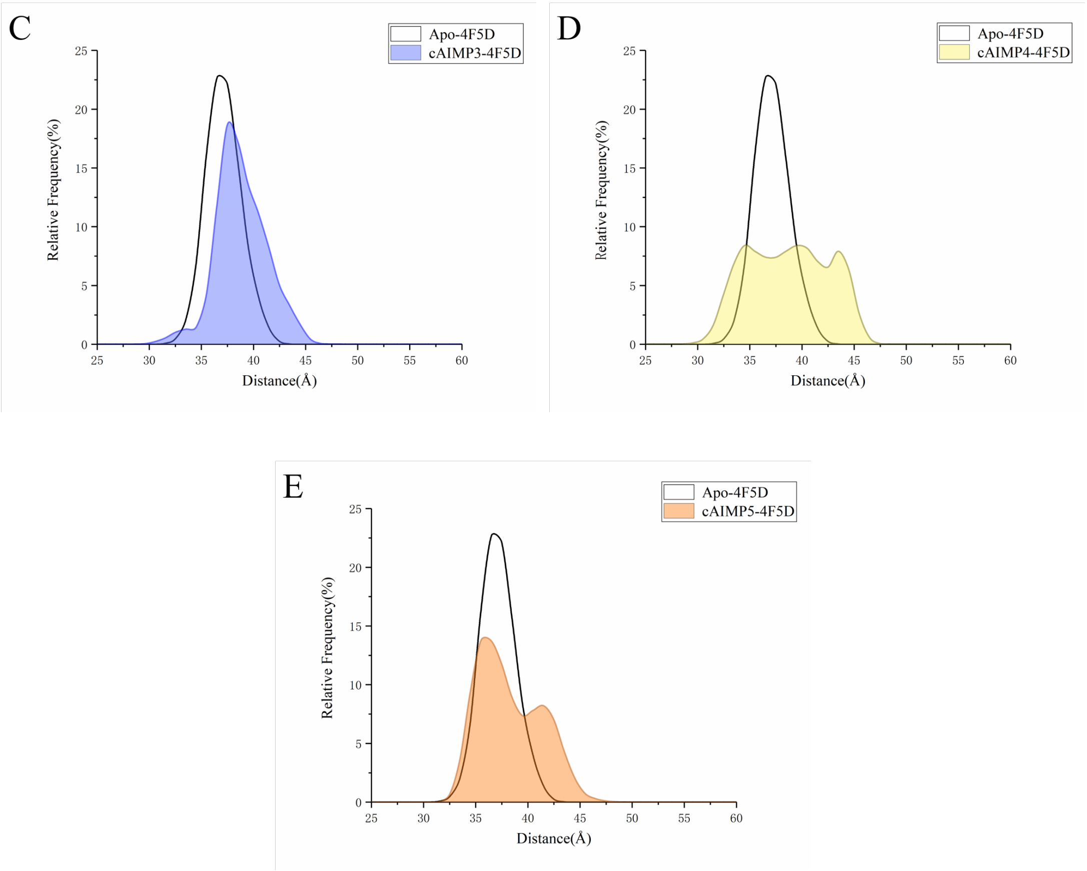

Figure S11. His185A–His185B distance distributions for all systems in closed state during the MD simulations.

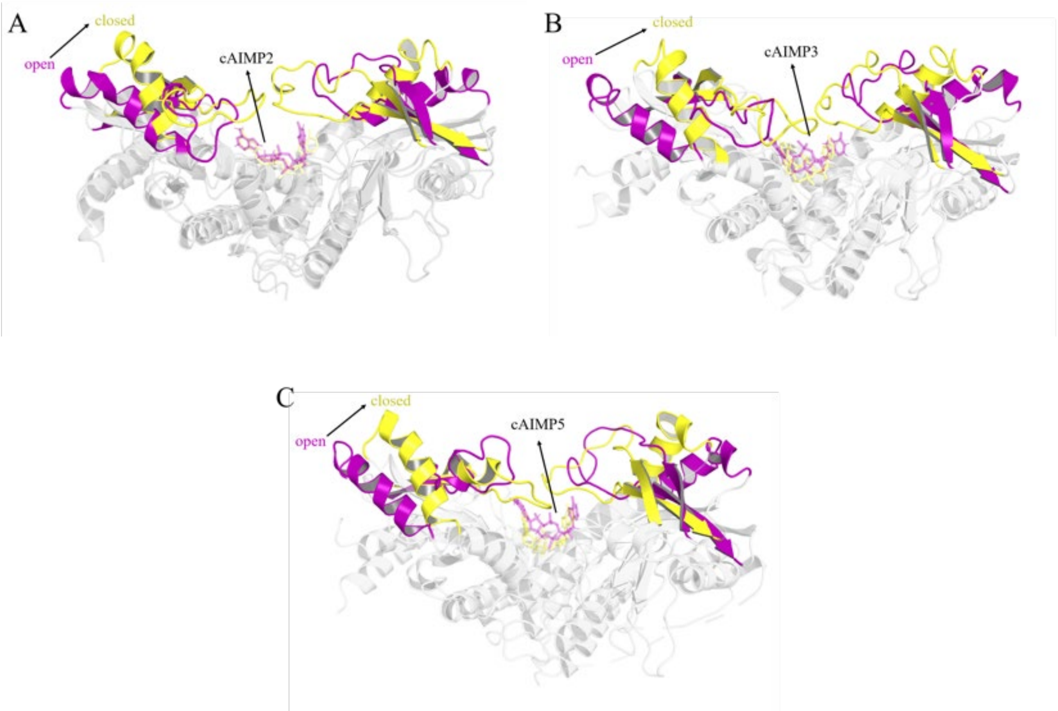

Figure S12. Open-to-closed conformational changes during the MD simulations of (A) cAIMP2-4F5Y (purple color: the structure extracted at 51 ns, yellow color: the structure extracted at 209 ns), (B) cAIMP3-4F5Y (purple color: the structure extracted at 84 ns, yellow color: the structure extracted at 212 ns), and (C) cAIMP5-4F5Y (purple color: the structure extracted at 33 ns, yellow color: the structure extracted at 187 ns).
